# Developmental protein kinase C hyper-activation results in microcephaly and behavioral abnormalities in zebrafish

**DOI:** 10.1038/s41398-018-0285-5

**Published:** 2018-10-23

**Authors:** Taian Liu, Yujian Shi, Matthew T. V. Chan, Gang Peng, Quan Zhang, Xiao Sun, Zeyao Zhu, Yuxin Xie, Kathy W. Y. Sham, Jianzhen Li, Xiaodong Liu, Idy H. T. Ho, Tony Gin, Zhonghua Lu, William K. K. Wu, Christopher H. K. Cheng

**Affiliations:** 1 Brain Cognition and Brain Disease Institute (BCBDI), Shenzhen Institutes of Advanced Technology, Chinese Academy of Sciences, Shenzhen, China; 2 Department of Anaesthesia and Intensive Care, The Chinese University of Hong Kong, Hong Kong, China; 3 School of Biomedical Sciences, The Chinese University of Hong Kong, Hong Kong, China; 4 Institutes of Brain Science, State Key Laboratory of Medical Neurobiology and Collaborative Innovation Center for Brain Science, Fudan University, Shanghai, China; 5 State Key Laboratory of Digestive Diseases, Institute of Digestive Diseases and Li Ka Shing Institute of Health Sciences, The Chinese University of Hong Kong, Hong Kong, China; 6 CUHK Shenzhen Research Institute, Shenzhen, China

## Abstract

Susceptible genetic polymorphisms and altered expression levels of protein kinase C (PKC)-encoding genes suggest overactivation of PKC in autism spectrum disorder (ASD) development. To delineate the pathological role of PKC, we pharmacologically stimulated its activity during the early development of zebrafish. Results demonstrated that PKC hyper-activation perturbs zebrafish development and induces a long-lasting head size deficit. The anatomical and cellular analysis revealed reduced neural precursor proliferation and newborn neuron formation. β-Catenin that is essential for brain growth is dramatically degraded. Stabilization of β-catenin by gsk3β inhibition partially restores the head size deficit. In addition, the neuropathogenic effect of developmental PKC hyper-activation was further supported by the alterations in the behavioral domain including motor abnormalities, heightened stress reactivity and impaired habituation learning. Taken together, by causally connecting early-life PKC hyper-activation to these neuropathological traits and the impaired neurogenesis, these results suggest that PKC could be a critical pathway in ASD pathogenesis.

## Introduction

Autism spectrum disorder (ASD) is a group of heterogeneous disorders with varying degrees of social, communicative and behavioral problems^1^. ASD is diagnosed by two or more core behavioral features: (1) impaired social interaction and communication and (2) repetitive, restrictive behaviors^2,3^. Other neurological abnormalities are also frequently observed in ASD such as abnormal brain growth, sensory problems, intellectual disability, motor deficits and epilepsy^4–6^. Despite the facts that ASD is highly heritable and has strong genetic component, a growing body of evidence suggests the genetic susceptibility might combine with environmental insults to shape individual’s risk to develop ASD^2,7–10^. However, whether they converge into common molecular pathways is now a hot point of speculation.

Protein kinase C (PKC), a group of serine/threonine-related protein kinases, represents a potential molecular pathway mediating both genetic and environmental risk factors for ASD. These enzymes can be classified into three subgroups: (1) conventional PKC isoforms (PKC-α, -β1, -β2 and -γ) that are activated by calcium and diacylglycerol (DAG) or phorbol esters like phorbol 12-myristate 13-acetate (PMA); (2) novel PKCs (PKC-δ, -ε, -θ and -η) that are activated by DAG or PMA, but not by calcium; and (3) atypical PKCs (PKC-ζ and -ι/λ) that are insensitive to calcium, DAG or PMA^11,12^.

PKC was first linked to ASD by a genetic association study of *PRKCB1*. The haplotypes of three single-nucleotide polymorphisms (SNPs) within *PRKCB1* locus are found to be strongly associated with autism^13^. They were further replicated in another independent study^14^. Recent exome sequencing for ASD has also identified a de novo functional mutation in *PRKCA* gene locus^15^. In addition to the genetic findings, multiple lines of evidence suggest the down-regulation of PKC isozymes like *PRKCB1*, *PRKCE* and *PRKCG* and the overall PKC activity in autistic brains^16–19^. Remarkably, more comprehensive analysis reveals an uncoordinated expression pattern between *PRKCB1* and several PKCβ-dependent genes involved in immune functions and oxidative stress^14^. Since immune activation and oxidative stress are deeply involved in the pathogenesis of ASD^20^, it suggests that the reduced expression of *PRKCB1* may be a negative feedback to buffer against these two pathogenic processes in autistic subjects^14,21^. This notion was favored by the identification of two SNPs that inactivate this negative feedback and increase the risk of ASD^14^. Therefore, it seems that the combination of genetic mutations and external disturbance is to excessively stimulate PKC, given that PKC is responsive to a variety of environmental risk factors for ASD such as maternal immune activation and stress^22–24^.

Thus, to explore the role of PKC in ASD development, we generated an animal model that mimics PKC-driven neuropathogenesis. In this respect, zebrafish provides a valuable tool to model this process because its in vitro embryo development allows specific modulation of PKC activity of larval fish using chemical PKC activator^25–27^, without the confounding maternal effects inevitable in mammalian models. As a result, we found that zebrafish subject to early-life chemically induced PKC activation display severe neurogenic defects as well as behavioral alterations. These findings suggest that PKC could be a critical pathway in ASD pathogenesis and provide insight into how PKC hyper-activation may affect the neurodevelopmental process.

## Materials and methods

### Zebrafish

AB strain zebrafish (*Danio rerio*) were used and maintained under 14 h light and 10 h dark cycles in circulating freshwater aquaria at 26 °C–28 °C. Fish were fed twice daily with newly hatched brine shrimp (Brine Shrimp Direct). Fish experiments were conducted following the regulations of the Animal Experimentation Ethics Committee of The Chinese University of Hong Kong and Institutional Animal Care and Use Committee of Fudan University.

Tg(Huc:GFP) has been reported previously^28^. For production of the same transgenic zebrafish, Tol2-elavl3-GCaMP6s^29^ obtained from Professor Ahrens was reconstructed by replacing GCaMP6s by green fluorescent protein (GFP) gene. Tg(Huc:GFP) transgenic embryos were generated by microinjection of plasmid DNA into single-cell embryos as described elsewhere^28,29^.

### Chemicals and treatment

PMA (Cat. No. 1201) and Go6983 (Cat. No. 2285) were purchased from Tocris Bioscience. Both drugs were dissolved in dimethyl sulfoxide (DMSO) and stored following the manufacturer’s instructions.

Zebrafish larvae were soaked in PMA or PMA+Go6983 diluted in fish water from 48 h post fertilization (hpf) to 72 hpf. Go6983 was added to the fish water 20 min prior to PMA treatment. After 72 hpf, the fish were washed extensively to remove the agents and then kept in normal condition. The concentrations of the chemicals used are specified in the text.

For the treatment of zebrafish embryos, chorions were removed by forceps at 12 hpf. Then, the dechorionated embryos were exposed to DMSO and PMA, respectively. After 24 hpf, the embryos were collected for subsequent analysis.

### RNA isolation and RT-qPCR

Total RNA samples were extracted from larval zebrafish using TRIzol Reagent (Life Technologies). Reverse transcription quantitative PCR (RT-qPCR) was carried out on ABI Viia 7 Real Time PCR System (Life Technology) by SYBR Green PCR Master Mix Kit (Life Technology) following standard protocols. The primers used are as follows.

*gabrb3*: AACTGGAATGCTGTGGATGGA/TGTCTATGCTCGCCACATCA

*gad1b*: CACGAGGAAACTGGGCATGA/AGTAGATCTCGCGCGAACAG

*gfap*: ACCCGTGACGGAGAGATCAT/ATGGCAGATCCTTCCTCTCC

*psd95*: GGAAGGGAAGAGCCAGTACG/GGTCGTGTCGTATGAGGGAC

*reln*: ATTTCCCCACATCCACCGAC/ TTACCGCTGTTGTAGGGCTG

*ef1a*: GGCTGACTGTGCTGTGCTGATTG/CTTGTCGGTGGGACGGCTAGG

*axin2*: AAGCCCCACAGCACTCAAAA/GGTCCTGAACAAAGGGGTGT

*jun*: CCCAAGAACGTGACGGATGA/ACGTGTTCAGATCCGCGTAA

*mycb*: ATACTCCGCCAAACAGTGGG/CATCTTCGTCATCGGATTCGC.

### Morphological analyses of zebrafish

Larval zebrafish were killed by MS-222 (Sigma) and images were taken on a Nikon SMZ 800 stereomicroscope. Body length refers to the distance between the tip of the snout and the end of the spine. Head size was defined by the otic vesicle and the semicircle of eyes as posterior and lower boundary, respectively^30^. For imaging of zebrafish brain, Tg(Huc:GFP)^28^ was used and images were acquired using Olympus SZX16 stereomicroscope with fluorescence imaging. The measurement was carried out by ImageJ software.

### Behavioral assays

As described previously^31^, larval fish were placed in a 24-well plate inside a wooden box. After habituation, larval activity was monitored on an automated video tracking system (Viewpoint, France) in two cycles of 5 min light and 5 min dark. The experiment was repeated for 3 times. The locomotion traces were analyzed in MATLAB (R2014a, The MathWorks) by a customized program.

### Western blotting

Lysates of larval zebrafish were separated by 10% sodium dodecyl sulfate–polyacrylamide gel electrophoresis. The separated proteins were transferred onto polyvinylidene fluoride membranes and immunoblotted with antibodies against β-catenin (Cat. No. YM3403, Immunoway) and α-tubulin (Cat. No. 24308, Rockland), respectively. The protein bands were visualized by a Western blotting kit (Millipore).

### Immunostaining and TUNEL labeling

Briefly, fish at 1 or 3 days post fertilization (dpf) were fixed in 4% paraformaldehyde (Sigma) overnight or for at least 2 days at 4 °C, rinsed with phosphate-buffered saline with Tween-20 (PBST), dehydrated and rehydrated in methanol–PBST sequentially. After digestion with proteinase K (20 μg/ml for 15 min), whole-mount immunostaining on larvae was carried out by pHH3 antibody (Cat. No. 3377, Cell Signaling). Whole-mount TUNEL (terminal deoxynucleotidyl transferase dUTP nick end labeling) labeling was performed using an in situ cell death detection kit (Ref. 11684795910, Roche). For immunostaining of dissected heads, the larvae after dehydration were embedded in paraffin and sectioned at 3.5 μm thickness. Sections were stained with β-catenin antibody (Cat. No. YM3403, Immunoway) together with 4′,6-diamidino-2-phenylindole (DAPI; Cat. No. 0100-20, Southern Biotech).

The images were taken on a confocal system with inverted microscope (Olympus FV1000). For whole-mount imaging, sets of images were taken along *z*-stack with a step of 5 μm, followed by visualization with maximum intensity projection. The dots were counted by ImageJ.

### EdU proliferation assays

The transgenic fish at 48 hpf was immersed with 500 μM 5-ethynyl-2’-deoxyuridin (EdU; Cat. No. C10640, ThermoFisher) solution for 20 min at room temperature and 1 h at 4 °C and subject to DMSO or PMA treatment, respectively. At 3 dpf, the fish was deyolked and digested with 0.25% trypsin (Cat. No. 25200056, ThermoFisher) into single cells. Then the EdU proliferation assay was performed according to the manufacturer’s protocols. Finally, zebrafish cells were analyzed by flow cytometry (BD LSRFortessa Cell Analyzer) using FITC and APC channels.

### Statistical analysis

Statistical analyses were performed using the GraphPad Instat software (GraphPad Software). All data were expressed as mean values ± SEM. Data were tested for significance using unpaired *t*-test, one-way analysis of variance (ANOVA) or two-way ANOVA that are specified in the figure legends.

## Results

### Early-life PKC hyper-activation leads to mild developmental delay and reduced brain size

The fact that the overall PKC activity is altered^19^ and several PKC isoforms belonging to either conventional or novel subgroups are dysregulated in autistic patients^16–18^ led us to speculate that multiple PKC isozymes may participate in ASD development. In order to generate a zebrafish model of PKC hyper-activation, we exposed larval fish to PMA to stimulate the overall PKC activity (except atypical PKCs)^27^. The aberrant PKC activation was confined to a critical period, namely from 48 hpf to 72 hpf, of zebrafish brain development which corresponds to that of initial primary neurogenesis in rodents^32^. This time window is frequently used for modeling ASD in rodents with environmental origins^33,34^.

**Fig. 1:**
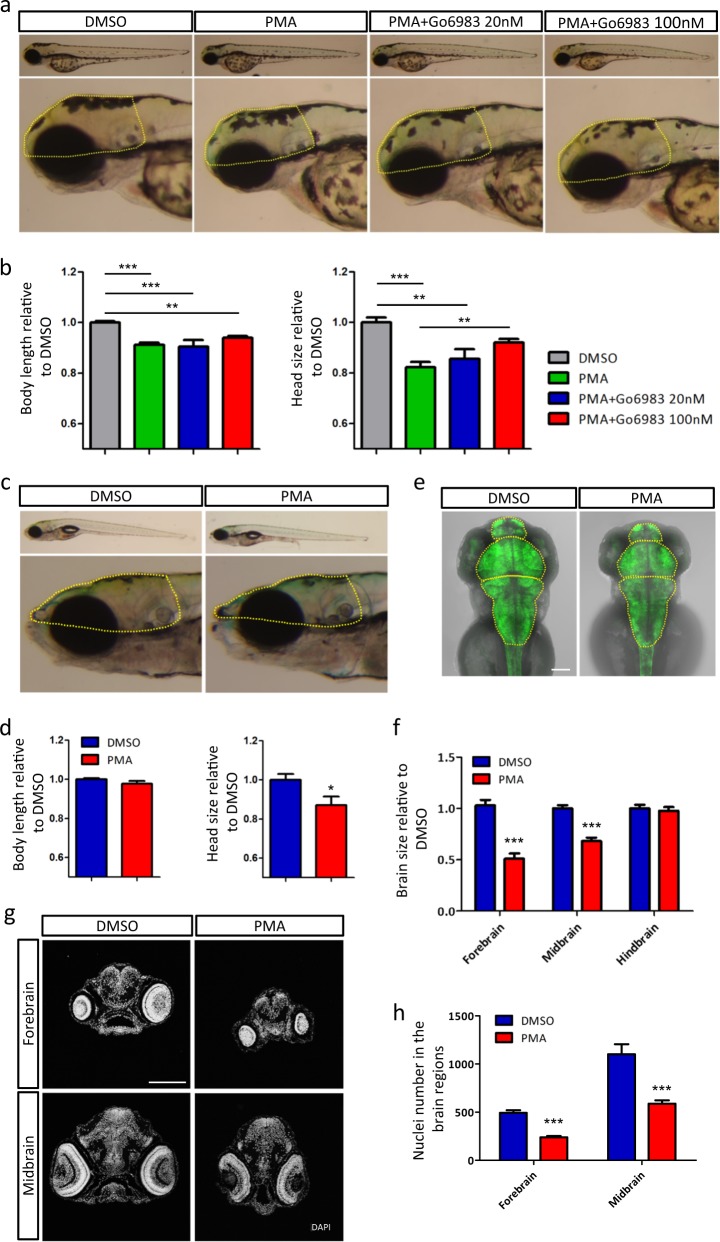
PKC overactivation during early development causes mild developmental delay and brain size deficit. **a** Lateral views of representative larvae (3 dpf) exposed to DMSO, PMA or PMA+Go6983. Head size was calculated with the boundary shown in yellow dotted line. **b** Quantitative representation of body length and head size (3 dpf) normalized to DMSO (DMSO *n* = 8, PMA *n* = 10, PMA+Go6983 20 nM *n* = 6, PMA+Go6983 100 nM *n* = 10, **p < 0.01, ***p < 0.001, one-way ANOVA). **c** Lateral views of representative larvae (9 dpf) previously exposed to DMSO or PMA. Head size was calculated with the boundary shown in yellow dotted line. **d** Quantitative representation of body length and head size (9 dpf) normalized to DMSO (DMSO *n* = 8, PMA *n* = 6, *p < 0.05, *t*-test analysis). **e** Dorsal views of zebrafish brains (3 dpf) visualized by Huc:GFP. Brain region was divided by yellow dotted line. Scale bar, 100 μm. **f** Quantitative representation of different brain regions normalized to DMSO (*n* = 16 per group, ***p < 0.001, *t*-test analysis). **g** DAPI staining of zebrafish head sections (3 dpf). Scale bar, 100 μm. **h** Nuclei number in forebrain and midbrain (*n* = 6 per group, ***p < 0.001, t-test)

To determine the neuropathological impact of PKC hyper-activation, we examined the whole-body expression levels of two neuron-specific genes, namely *reln* and *gad1b*, which are frequently found to be down-regulated in neuropsychiatric disorders including ASD^35–37^. A series of PMA dosages were tested. Only the concentration of 40 nM could induce a persistent down-regulation of *reln* and *gad1b* at 3 dpf (Supplementary Figure S1 and Table S1). A larger set of genes were also analyzed at an earlier time point. We found that early-life PMA challenge significantly attenuated the messenger RNA (mRNA) expression of these genes at 3 dpf (Supplementary Figure S1). We postulated that the overall reduction of neuron-specific gene expression might be associated with head size deficit. We therefore measured head size and body length of the zebrafish. It was revealed that developmental exposure to PMA significantly suppressed growth with 8.9% decrease in body length and 18.8% reduction in head size after PMA treatment (Fig. 1a, b). To validate the specificity of PMA, a broad-spectrum PKC inhibitor Go6983 was used to block the effect of PMA^38^. Treatment with Go6983 restored the head deficit but was not so effective in reversing the development delay of body growth (Fig. 1a, b). This discrepancy might be due to the incomplete overlap of the binding partners of these two agents. In particular, PKC-ε, -θ and -η are insensitive to Go6983^38^, and may be more important to zebrafish somatic trunk growth.

Gene expression data also imply that the head size deficit persisted at least to 6 dpf (Supplementary Figure S1). To confirm this speculation, we performed the measurement again at a later time point of 6 dpf. At 9 dpf, the head size remained aberrantly small but to a less extent, while no significant difference was observed in the body length and eye size (Fig. 1c, d; Supplementary Figure S2). The swim bladder was also well developed (Fig. 1c). Since the body length is generally used as an indicator of larval fish developmental stages^39^, these results suggested that exposure to PMA predominantly induced head size deficit.

Then, to examine the brain structure, a transgenic fish Tg (Huc:GFP) expressing GFP in post-mitosis neurons was used^28^. Results showed that both the forebrain and midbrain were adversely affected with reduction by 50.8% and 30.1% in size, respectively (Fig. 1e, f). However, alteration of the hindbrain was not obvious (Fig. 1e, f). Nucleus count of the head section indicated that cell number within the forebrain and midbrain regions decreased by 51.4% and 46.7% as compared with DMSO-exposed control (Fig. 1g, h). Overall, our results demonstrated that early-life PKC hyper-activation could cause mild developmental delay, small head size and decreased neural cell number in zebrafish.

### Impaired neurogenesis by PKC overactivation

**Fig. 2:**
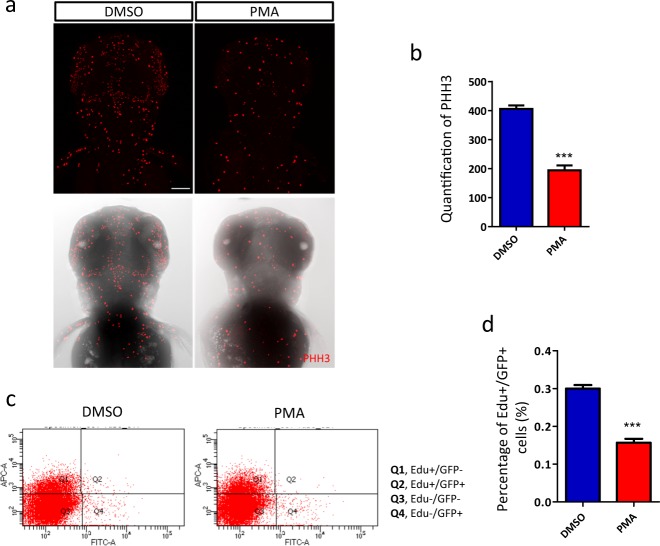
Aberrant PKC activation impaired neurogenesis. **a**, **b** Reduced mitotic cells in zebrafish head (3 dpf). **a** Fluorescence of pHH3-positive dots (the upper row) merged with brightfield images of zebrafish head (the lower row) (*n* = 26 per group, ***p < 0.001, *t*-test). Scale bar, 100 μm. **c** Flow cytometry analysis of the different populations of cells. **d** Newborn neurons (Q2) are significantly reduced in PMA-exposed fish (*n* = 9 per group, *p* < 0.001, *t*-test)

The reduced brain size and cell number suggest that PKC hyper-activation may cause neurogenic defects in zebrafish. Thus, we quantified the proliferating cells within zebrafish head. Phosphohistone-H3 (pHH3) staining of whole-mount fish revealed much less dividing cells in the head of PMA-exposed fish (Fig. 2a, b). A temporary elevation of apoptosis was also observed at 60 hpf (Supplemental Figure S3). These data indicate the neural precursor pool was decreased by PKC hyper-activation. However, it is unclear to what extent apoptosis may contribute to this process.

To confirm the negative impact of PKC hyper-activation on neurogenesis, a more direct examination was carried out by dual-labeling of the newborn neurons with GFP in Tg(Huc:GFP)^28^ and EdU that had been incorporated into synthesizing DNA of neural precursors beforehand. It is noteworthy that not all the neural precursors could take up EdU during a 90-min incubation. However, the number of dual-labeled cells was expected to be proportionate to the production of newborn neurons. Flow cytometry analysis revealed that the dual-labeled cells apparently decreased by 47.7% compared with the control group (Fig. 2c, d). These results strongly support that neurogenesis was greatly impaired by developmental PKC hyper-activation.

Although the massive neurogenesis begins from 2 dpf in zebrafish as noted earlier, there is a peak of production of transitory neurons around 24 hpf^32^. The effect of PKC hyper-activation on the embryonic neurogenesis was also evaluated. Results demonstrate that this wave of neurogenesis is less vulnerable to PKC stimulus as the proliferative and apoptotic cells within the head are less affected at this time point (Supplemental Figure S4).

### Excessive PKC signaling promotes degradation of β-catenin

**Fig. 3:**
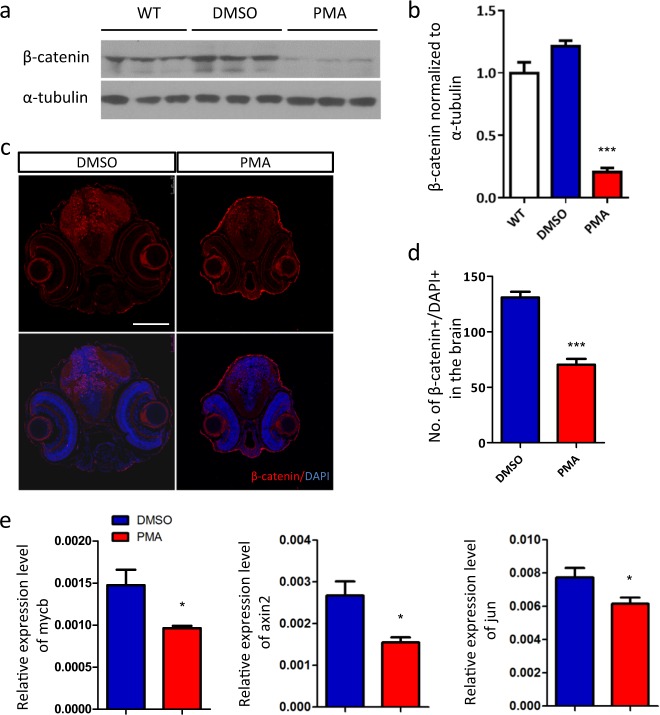
Excessive PKC signaling promotes the degradation of β-catenin. **a** Western blot analysis of β-catenin. **b** Quantification of the protein level of β-catenin relative to wild-type (WT) group (*n* = 3 per group, ***p < 0.001, *t*-test). **c** The nuclear accumulation of β-catenin is reduced by PMA treatment, **d** indicated by the quantification of β-catenin^+^/DAPI^+^ dots in brain region (*n* = 6 per group, ***p < 0.001, *t*-test). **e** RT-qPCR analysis of the transcriptional target of β-catenin, *mycb, axin2* and *jun* (*n* = 6 per group, *p < 0.05, *t*-test)

Two previous studies demonstrated that β-catenin is negatively regulated upon persistent PKC activation^40,41^. Importantly, deregulated Wnt/β-catenin signaling has been deeply implicated in aberrant brain development and autism^2,42–45^. Thus, we hypothesized that PKC hyper-activation could deregulate the Wnt/β-catenin pathway in our model. To test this hypothesis, we determined the whole-body protein level of β-catenin in wild-type, PMA- and DMSO-exposed fish. Concordantly, β-catenin was sharply down-regulated by developmental PKC hyper-activation (Fig. 3a, b), with no obvious change at transcriptional level (data not shown), indicating increased β-catenin protein degradation. We also stained β-catenin in transverse head sections to determine its expression in neural tissues. Only β-catenin co-localized with DAPI was counted to reflect the nuclear accumulation of this transcription factor. A significant decrease in double-positive dots was observed in PMA-exposed fish (Fig. 3c, d).

To confirm the disruption of Wnt/β-catenin pathway upon PMA exposure, we examined the expression of several well-known transcriptional targets of β-catenin, *mycb*^46,47^, *axin2*^48^ and *jun*^49^. As expected, the transcription of these three genes were all reduced, supporting the down-regulation of Wnt/β-catenin signaling (Fig. 3e). Collectively, our data revealed in PMA-exposed fish a disrupted β-catenin signaling which might be involved in PKC hyper-activation-induced neuropathogenesis.

### Stabilizing of β-catenin by gsk3β inhibition partially restores the head size deficit

**Fig. 4:**
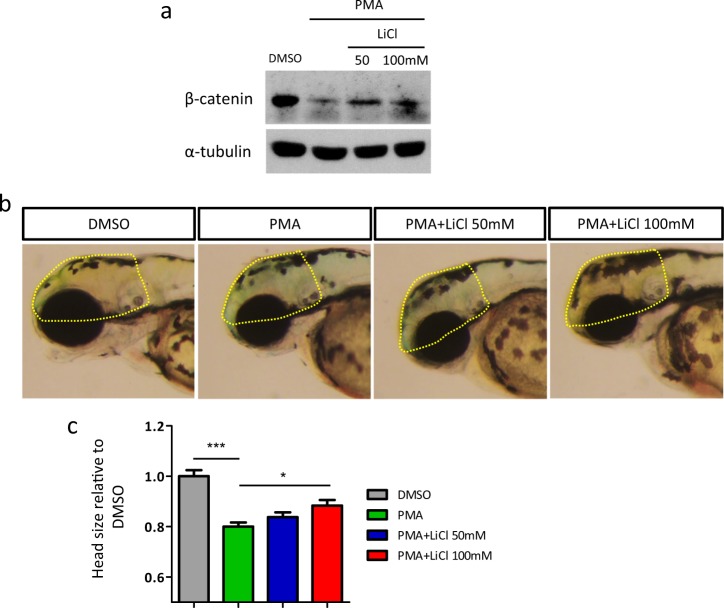
Stabilizing of β-catenin by gsk3β inhibition partially restores the head size deficit. **a** Western blot analysis of β-catenin. **b** Lateral views of representative larvae (3 dpf) exposed to DMSO, PMA or PMA+LiCl. Head size was calculated with the boundary shown in yellow dotted line. **c** Quantitative representation of body length and head size (3 dpf) normalized to DMSO (DMSO *n* = 14, PMA *n* = 18, PMA+LiCl 50 mM *n* = 12, PMA+LiCl 100 mM *n* = 18, *p < 0.05, ***p < 0.001, one-way ANOVA)

To test whether β-catenin acts downstream of PKC to induce microcephaly, lithium chloride (LiCl) that inhibits glycogen synthase kinase-3β (gsk3β) was used to stabilize β-catenin^50^. Since PKC parallels to gsk3β in promoting the degradation of β-catenin by sharing similar phosphorylation sites^40,50^, LiCl treatment could only partially restore the protein level of β-catenin (Fig. 4a). Nevertheless, we found that reduced β-catenin degradation could apparently attenuate the head size deficit in PMA-exposed fish (Fig. 4b, c). It consolidates our finding that β-catenin is an important mediator of PKC in perturbing brain growth.

### Behavioral abnormalities of zebrafish with developmental PKC hyper-activation

**Fig. 5:**
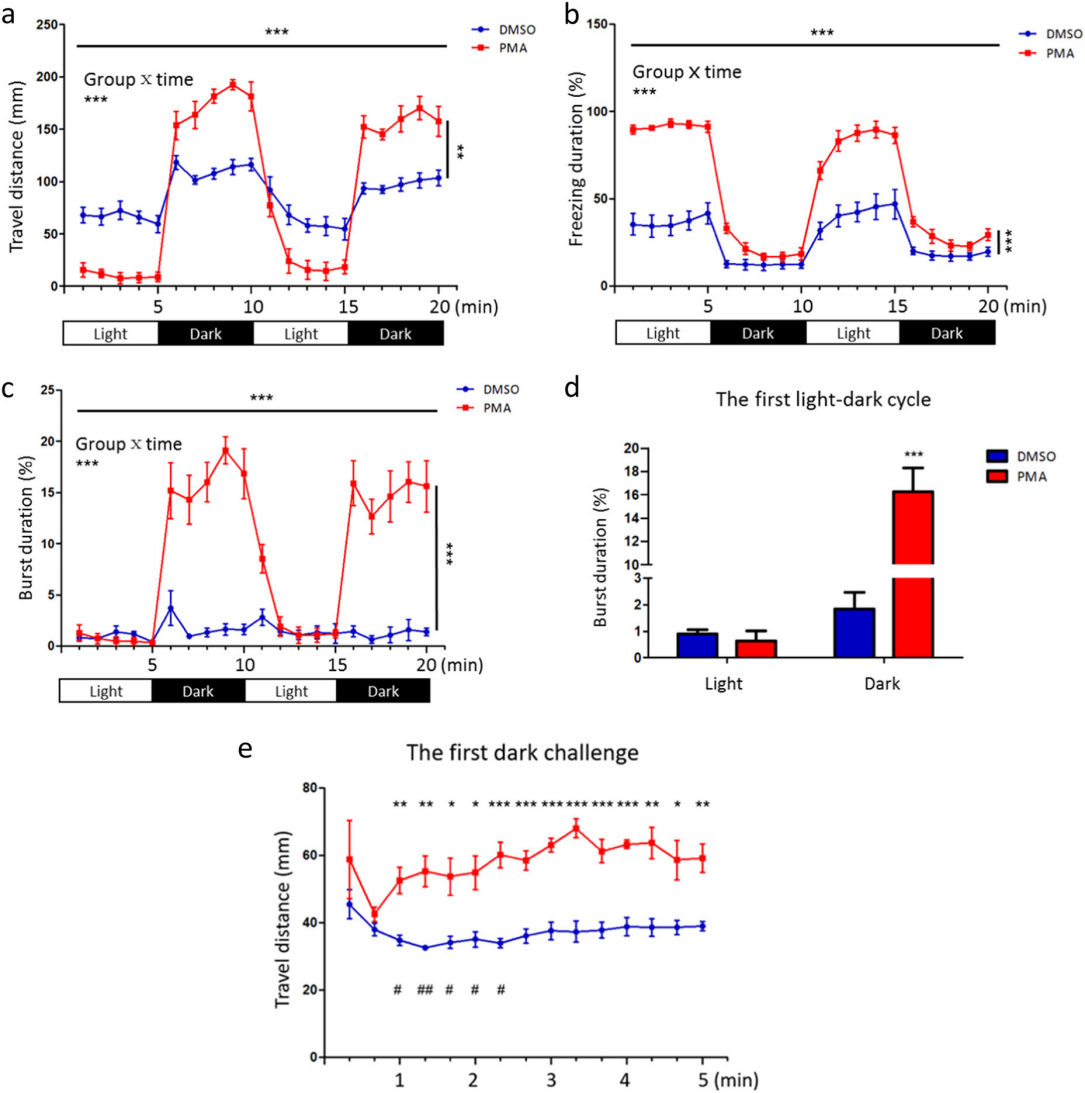
Developmental PKC overactivation leads to hypo-activity, exaggerated stress response to visual stimuli and impaired habituation learning. **a** Zebrafish early exposed to PMA are hypoactive in the light/normal phase but hyperactive in the dark phase as revealed by the travel distance (mm) per minute. **b** Hypo-activity in the light phase is caused by prolonged freezing duration. Freezing refers to movement with a speed lower than 0.57 mm per second. **c** Hyperactivity in the dark phase is attributed to the increased burst duration. Burst refers to movement with a speed exceeding 5.7 mm per second. **a**–**c** Significant effects of time (horizontal lines), group (vertical lines) and group × time interactions are shown (*n* = 48 for DMSO group, *n* = 44 for PMA group; **p < 0.01, ***p < 0.001, two-way ANOVA). **d** PMA-exposed fish show much longer burst duration in the dark phase but no significant difference in the light phase compared with the control group during the first light–dark cycle (*n* = 48 for DMSO group, *n* = 44 for PMA group; ***p < 0.001, *t*-test). **e** Impaired habituation learning is responsible for the elevated stress response to photic change in the PMA-exposed group (*n* = 48 for DMSO group, *n* = 44 for PMA group; *p < 0.05, **p < 0.01, ***p < 0.001, *t*-test for comparison between DMSO and PMA groups at each time point; ^#^*p* < 0.05, ^##^*p* < 0.01, one-way ANOVA for comparison of each time point with the first one in the control group)

Since the final readout of neuropathological condition is behavioral alterations, we assessed the behavioral changes of PMA-exposed zebrafish at 5 dpf, at which stage robust and measurable behaviors had been developed^51^. The locomotion activity in light/dark cycles represents one of the most extensively used endpoints to evaluate the escape or avoidance behaviors of zebrafish as well as its motor ability as larval zebrafish before 2 weeks post fertilization is aversive to darkness^51,52^. We observed a very low swimming speed of PMA-exposed fish in the light phase, of about 16% relative to DMSO-exposed group (Fig. 5a and Supplemental Figure S5). This was attributed to an extremely long freezing duration, in which the fish spent more than 90% of the time (Fig. 5b and Supplemental Figure S5). Since the tested fish had been acclimated in the plate for at least 3 h, the increased freezing was more likely to be an outcome of motor abnormalities.

In the dark phase, the fish were stressed and displayed escape/avoidance behaviors though high-speed movement^51,52^. The activity of both the PMA- and DMSO-exposed groups increased abruptly (Fig. 5a and Supplemental Figure S5). Surprisingly, the average speed of PMA-exposed fish became even much higher than the DMSO-exposed group with a sharply prolonged burst duration (Fig. 5a, c, d). However, the burst swimming of DMSO-exposed group did not increase significantly (Fig. 5c, d) with the increased movement distance primarily arising from the shortened freezing time (Fig. 5b and Supplemental Figure S5). At the same time, the freezing behaviors of PMA-exposed group declined acutely to a slightly higher level than the DMSO-exposed group (Fig. 5b and Supplemental Figure S5). Then, we select the total burst duration to quantify the difference in stress response between two groups during dark challenge because this value appears to be invulnerable to motor problems seen in PMA-exposed fish (Fig. 5d). It shows that PMA-exposed group has about 9 time longer burst duration than DMSO-exposed control during the dark phase (Fig. 5d), suggesting developmental PKC hyper-activation rendered the fish over-responsive to the decrease of light intensity. In addition, our analysis on the second light–dark cycle leads to a similar conclusion (Fig. 5a; Supplemental Figure S5, S6a).

We next examined the reasons behind the heightened stress response. Interestingly, we found no significant difference between the two groups in the locomotion distance at the very beginning of stress condition (Fig. 5e). However, the difference became pronounced at later time points because there was apparent decrease following the peaks generated immediately after the light-off in the control group (Fig. 5e). This is a process of habituation, a fundamental form of non-associated learning in animals^53,54^. However, it was impaired in the PMA-exposed fish, as no sharp decline in activity was observed throughout the first dark phase (Fig. 5e). We also note that there was a habituation process between the two dark phases as shown in the control fish but not in the PMA-exposed group. It decreased the initial response of the DMSO-exposed fish to the second dark challenge (Supplementary Figure S6b). As a result, no obvious decline of activity was observed in the second dark phase (Fig. 5a). In summary, we found zebrafish with developmental PKC hyper-activation displayed atypical behaviors including hypo-activity and exaggerated stress response to visual stimuli arising from the impaired habituation learning.

## Discussion

The development of possible therapeutic strategies for autism relies on the identification of targetable molecular pathways underlying neurodevelopmental defects. The present study demonstrates that sole PKC hyper-activation during early development could cause many pathological features such as developmental delay, smaller size of heads and eyes, motor abnormalities and exaggerated stress response. In fact, it is not surprising that exposure to PMA could hamper the development of larval zebrafish. However, it should be noted that the neurodevelopmental deficit is not just a result of the gross development delay. For example, both the head and body development were adversely affected by early PKC stimulation (Fig. 1a, b), but the somatic trunk and eye size deficit recovered much faster afterwards with no other obvious organ defects observed (Fig. 1c, d; Supplemental Figure S2). In contrast, the negative effects on brain development persisted (Fig. 1c, d), which is further supported by the altered neurobehavioral parameters (Fig. 5). These data fit well with the proposed developmental vulnerability of the neural system, in which the highly orchestrated brain development is less tolerant to the deleterious genetic mutations or external disturbance^20,55^. This notion also corresponds with the clinical investigations that children with ASD often bear multi-organ developmental problems with dominant dysfunction in the brain^20,56^. In addition, our findings that PKC hyper-activation primarily perturbed the growth of the forebrain and midbrain may partly explain the disproportionately affected brain regions in microcephaly, in which the impact on the cerebellum is not as big as that on the forebrain and midbrain^57^.

Our findings further suggest that PKC hyper-activation may perturb the brain growth through disrupting β-catenin-regulated neurogenesis. Since zebrafish cells double rapidly during early development, a big reduction of proliferative cells and total nucleus count were observed within the head of PMA-exposed fish. This sharp decrease of neural precursor proliferation and slighter changes of apoptosis also consists with the conception that the major role of β-catenin during neurogenesis is to stimulate cell proliferation (Fig. 2 and Supplementary Figure S3)^58–60^.

Actually, dysregulated Wnt/β-catenin pathway^43,44^ and neurogenesis^2,61–63^ are frequently proposed in the pathophysiology of ASD. Mirror extreme phenotypes, both macrocephaly and microcephaly, have been reported in autistic patients^63–65^. Our data contribute to a mechanistic understanding of the role of PKC in ASD progression by establishing a causal link between PKC activation and the impairment of β-catenin-regulated neurogenesis. However, it should be noted that PKC might also predispose zebrafish brain to abnormal functions through other processes such as affecting neuron dendritic spine strength and phosphorylation of neuroligin-4X which is another autism risk molecule^24,66,67^.

In addition, the neuropathogenic effect of developmental PKC hyper-activation was supported by behavioral alterations. One neurobehavioral consequence of early-life PKC hyper-activation is hypo-activity. And this hypo-activity may not be due to defect in muscle contraction because high-speed movement can be achieved in stress condition (Fig. 5a). It may parallel to the motor control problems seen in most ASD patients^2,68^. Apart from motor abnormalities, we identified an elevated stress reactivity in PMA-exposed fish arising from impaired habituation learning, suggesting the fish may be hypersensitive to the photic stimuli (Fig. 5a, d, e). It has striking relevance to ASD because the sensory problems, primarily hypersensitivity caused by reduced habituation, are estimated to affect about 90% of individuals with ASD^69,70^. Further studies are highly warranted to explore the neuronal basis of these ASD-related behaviors.

However, we should also note that pharmacological manipulation of PKC in this study, while easier to control the duration and magnitude of PKC activation, has several limitations. Firstly, it failed to discriminate the contributions of different PKC isoforms. Secondly, although the concentration of PMA used in our study is much less than the cell line experiments, it still caused a death rate of 24% (Supplemental Table S1). It may be due to the vulnerability of the developing organs. Specific manipulation of PKC isoforms in the brain, or even certain cell types, is required in the future experiments to provide a more precise interpretation of the role of PKC in neurodevelopmental disorders.

Nevertheless, our study established a causal link between developmental PKC hyper-activation and the neuropathological traits consistent with that of ASD. It may also shed new insight into the molecular mechanism by which predisposing PKC gene variants and some environmental factors exert their pathogenic actions.

## Electronic supplementary material

Supplemental Table S1

Supplemental Figure S1

Supplemental Figure S2

Supplemental Figure S3

Supplemental Figure S4

Supplemental Figure S5

Supplemental Figure S6
