# Supplementary material for: Developmental protein kinase C hyper-activation results in microcephaly and behavioral abnormalities in zebrafish
Source: Transl Psychiatry. 2018 Oct 23;8:232. doi: 10.1038/s41398-018-0285-5 (PMC6199330; doi:10.1038/s41398-018-0285-5)
Supplement: Supplementary file 1 — Supplemental Table S1 [file 41398_2018_285_MOESM1_ESM.docx]

| Concentration (nM) | 0 | 20 | 40 | 50 |
| --- | --- | --- | --- | --- |
| Survival rate* (%) | 96.8 | 98.1 | 76 | 51.4 |

**Table S1.** The effect of different concentrations of PMA on the survival rate of larval zebrafish

*Without obvious malformation
