# Supplementary material for: Developmental protein kinase C hyper-activation results in microcephaly and behavioral abnormalities in zebrafish
Source: Transl Psychiatry. 2018 Oct 23;8:232. doi: 10.1038/s41398-018-0285-5 (PMC6199330; doi:10.1038/s41398-018-0285-5)
Supplement: Supplementary file 2 — Supplemental Figure S1 [file 41398_2018_285_MOESM2_ESM.pptx]

## Slide 1
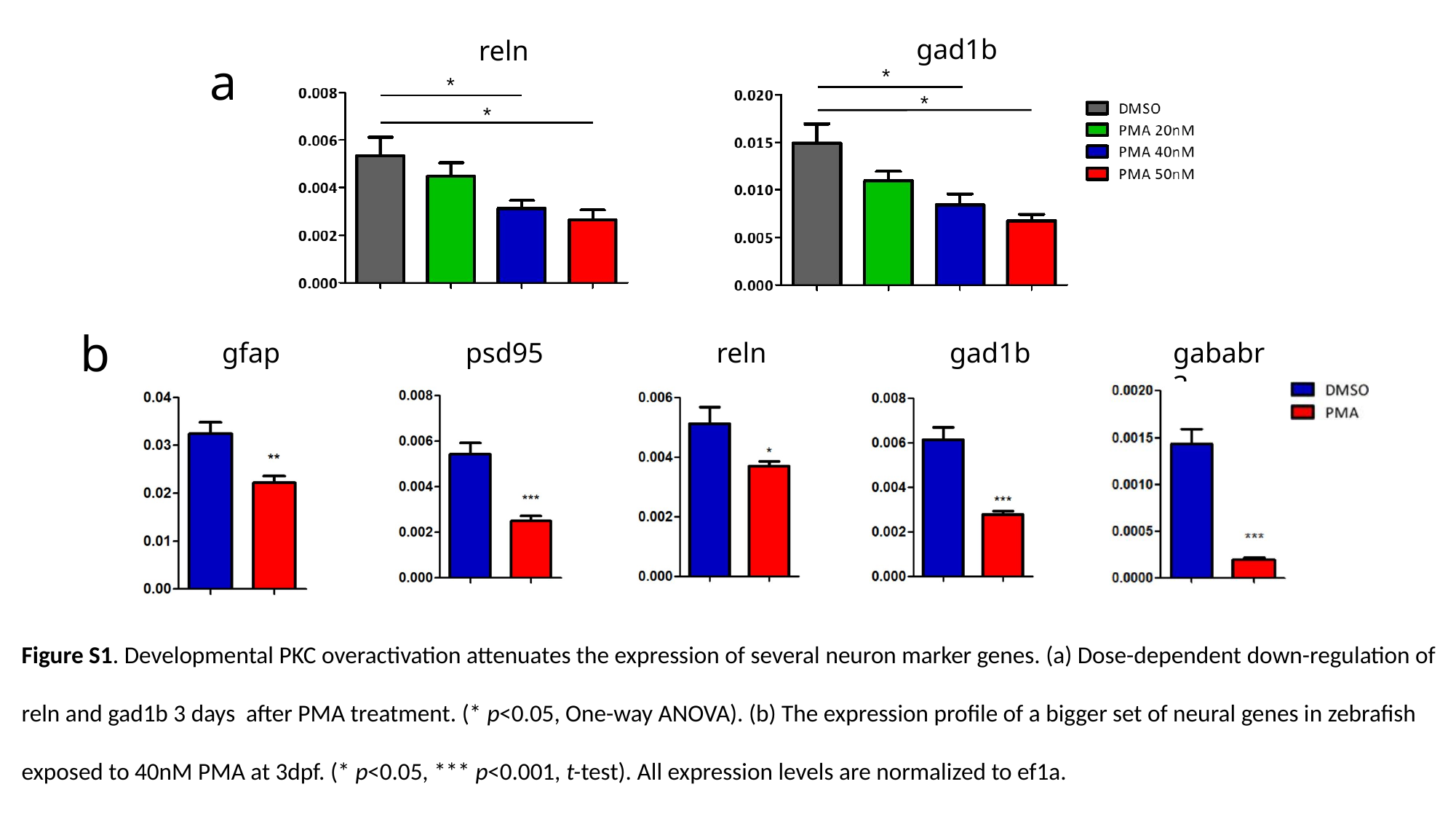

gad1b
reln
a
*
*
*
*
b
gfap
psd95
reln
gad1b
gababr3
Figure S1. Developmental PKC overactivation attenuates the expression of several neuron marker genes. (a) Dose-dependent down-regulation of reln and gad1b 3 days after PMA treatment. (* p<0.05, One-way ANOVA). (b) The expression profile of a bigger set of neural genes in zebrafish exposed to 40nM PMA at 3dpf. (* p<0.05, *** p<0.001, t-test). All expression levels are normalized to ef1a.
