# Supplementary material for: Developmental protein kinase C hyper-activation results in microcephaly and behavioral abnormalities in zebrafish
Source: Transl Psychiatry. 2018 Oct 23;8:232. doi: 10.1038/s41398-018-0285-5 (PMC6199330; doi:10.1038/s41398-018-0285-5)
Supplement: Supplementary file 3 — Supplemental Figure S2 [file 41398_2018_285_MOESM3_ESM.pptx]

## Slide 1
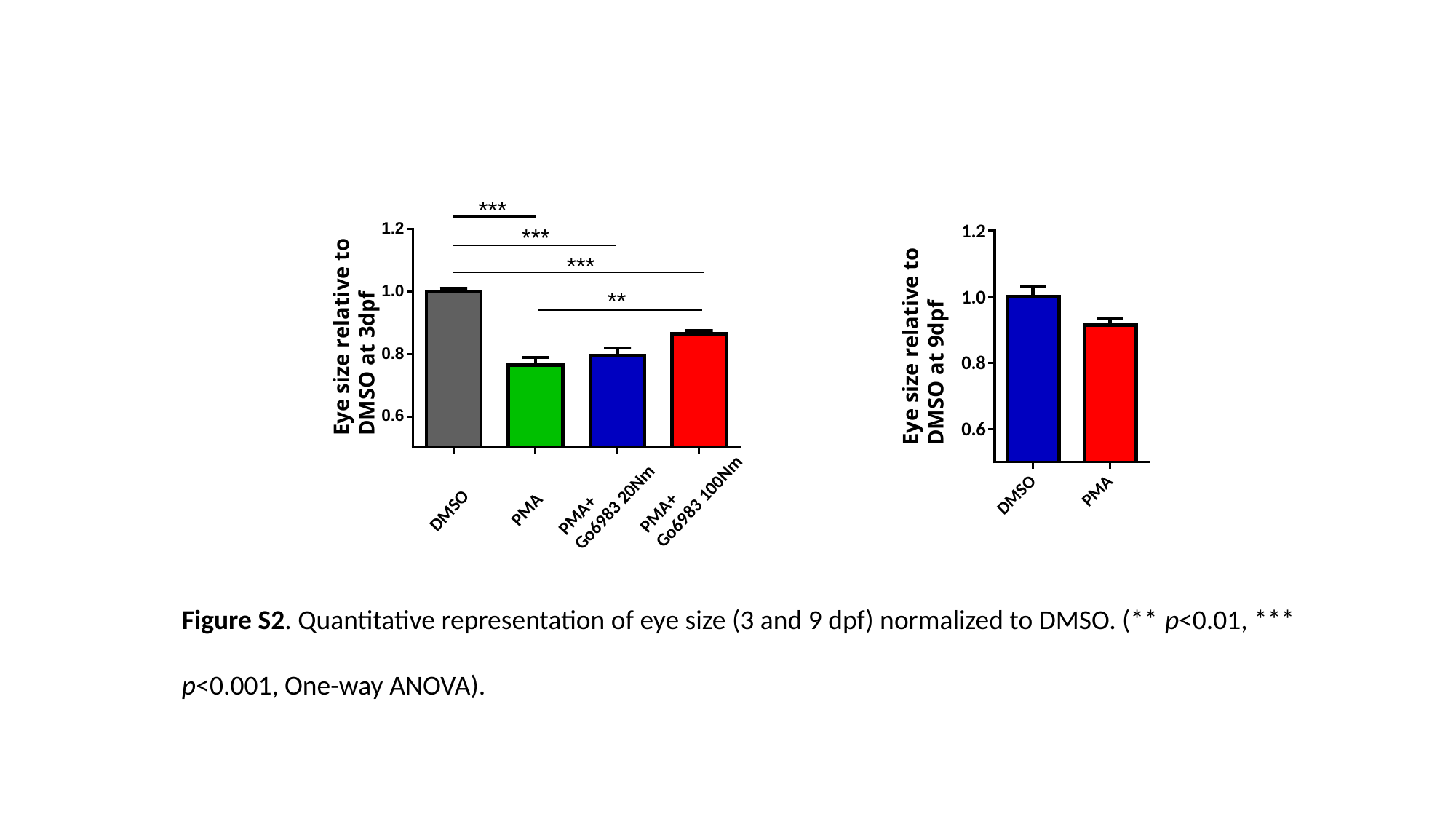

***
***
***
**
Eye size relative to DMSO at 3dpf
Eye size relative to DMSO at 9dpf
PMA
PMA+
Go6983 100Nm
DMSO
PMA+
Go6983 20Nm
Figure S2. Quantitative representation of eye size (3 and 9 dpf) normalized to DMSO. (** p<0.01, *** p<0.001, One-way ANOVA).
