# Supplementary material for: Developmental protein kinase C hyper-activation results in microcephaly and behavioral abnormalities in zebrafish
Source: Transl Psychiatry. 2018 Oct 23;8:232. doi: 10.1038/s41398-018-0285-5 (PMC6199330; doi:10.1038/s41398-018-0285-5)
Supplement: Supplementary file 4 — Supplemental Figure S3 [file 41398_2018_285_MOESM4_ESM.pptx]

## Slide 1
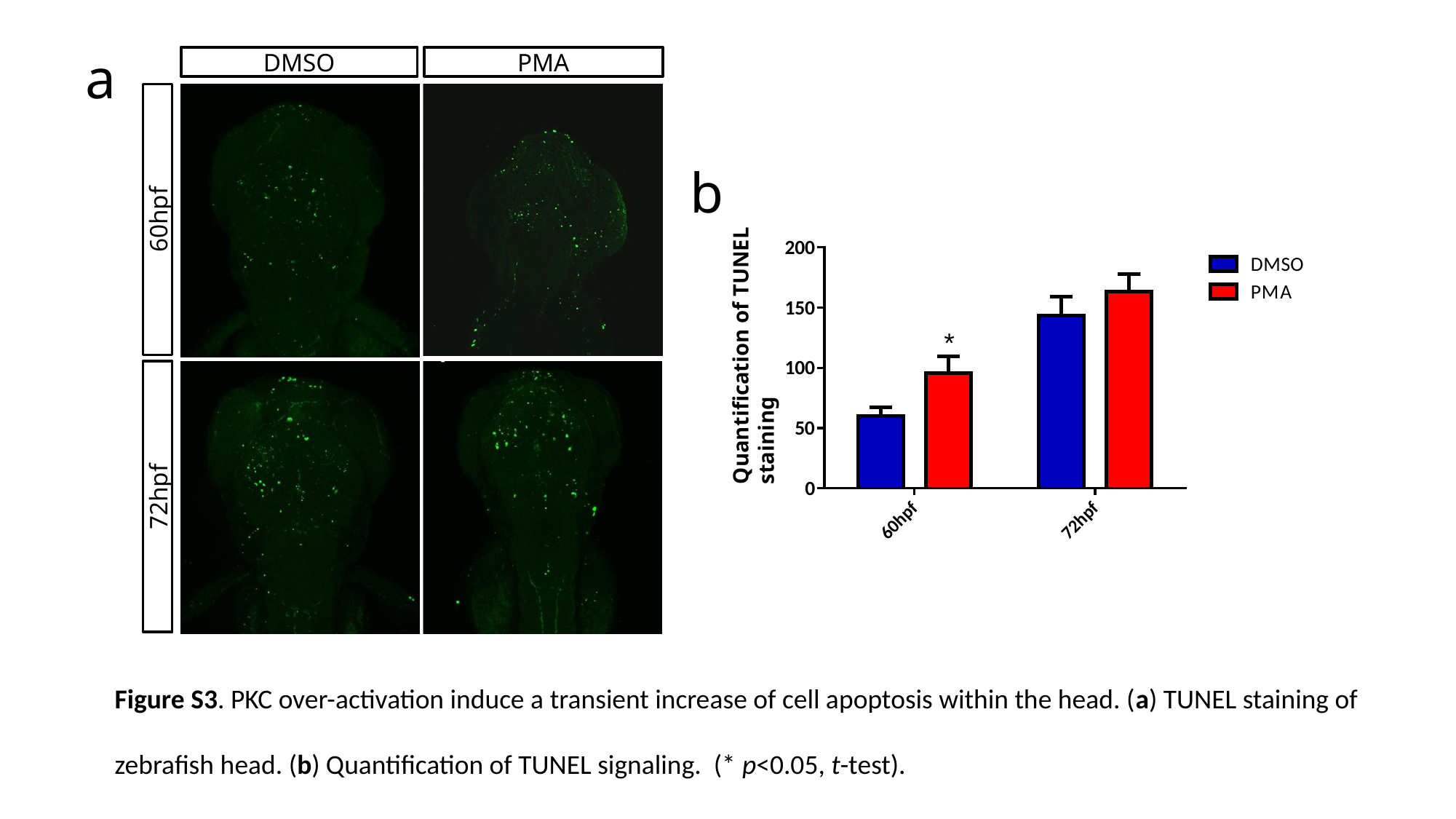

a
DMSO
PMA
b
60hpf
Quantification of TUNEL staining
*
72hpf
Figure S3. PKC over-activation induce a transient increase of cell apoptosis within the head. (a) TUNEL staining of zebrafish head. (b) Quantification of TUNEL signaling. (* p<0.05, t-test).
