# Supplementary material for: Developmental protein kinase C hyper-activation results in microcephaly and behavioral abnormalities in zebrafish
Source: Transl Psychiatry. 2018 Oct 23;8:232. doi: 10.1038/s41398-018-0285-5 (PMC6199330; doi:10.1038/s41398-018-0285-5)
Supplement: Supplementary file 5 — Supplemental Figure S4 [file 41398_2018_285_MOESM5_ESM.pptx]

## Slide 1
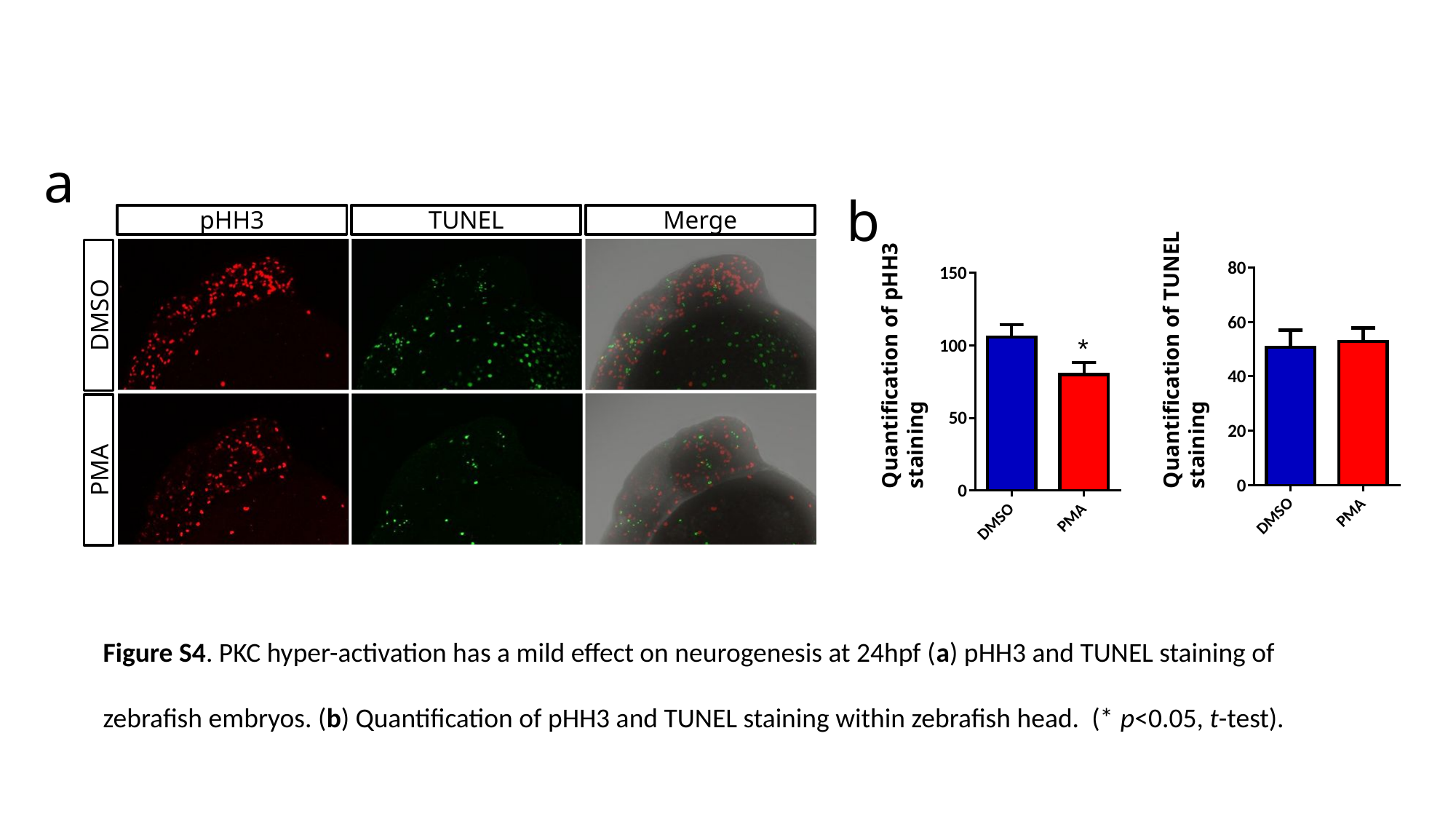

a
b
pHH3
Merge
TUNEL
DMSO
Quantification of pHH3 staining
Quantification of TUNEL staining
*
PMA
Figure S4. PKC hyper-activation has a mild effect on neurogenesis at 24hpf (a) pHH3 and TUNEL staining of zebrafish embryos. (b) Quantification of pHH3 and TUNEL staining within zebrafish head. (* p<0.05, t-test).
