# Supplementary material for: Developmental protein kinase C hyper-activation results in microcephaly and behavioral abnormalities in zebrafish
Source: Transl Psychiatry. 2018 Oct 23;8:232. doi: 10.1038/s41398-018-0285-5 (PMC6199330; doi:10.1038/s41398-018-0285-5)
Supplement: Supplementary file 6 — Supplemental Figure S5 [file 41398_2018_285_MOESM6_ESM.pptx]

## Slide 1
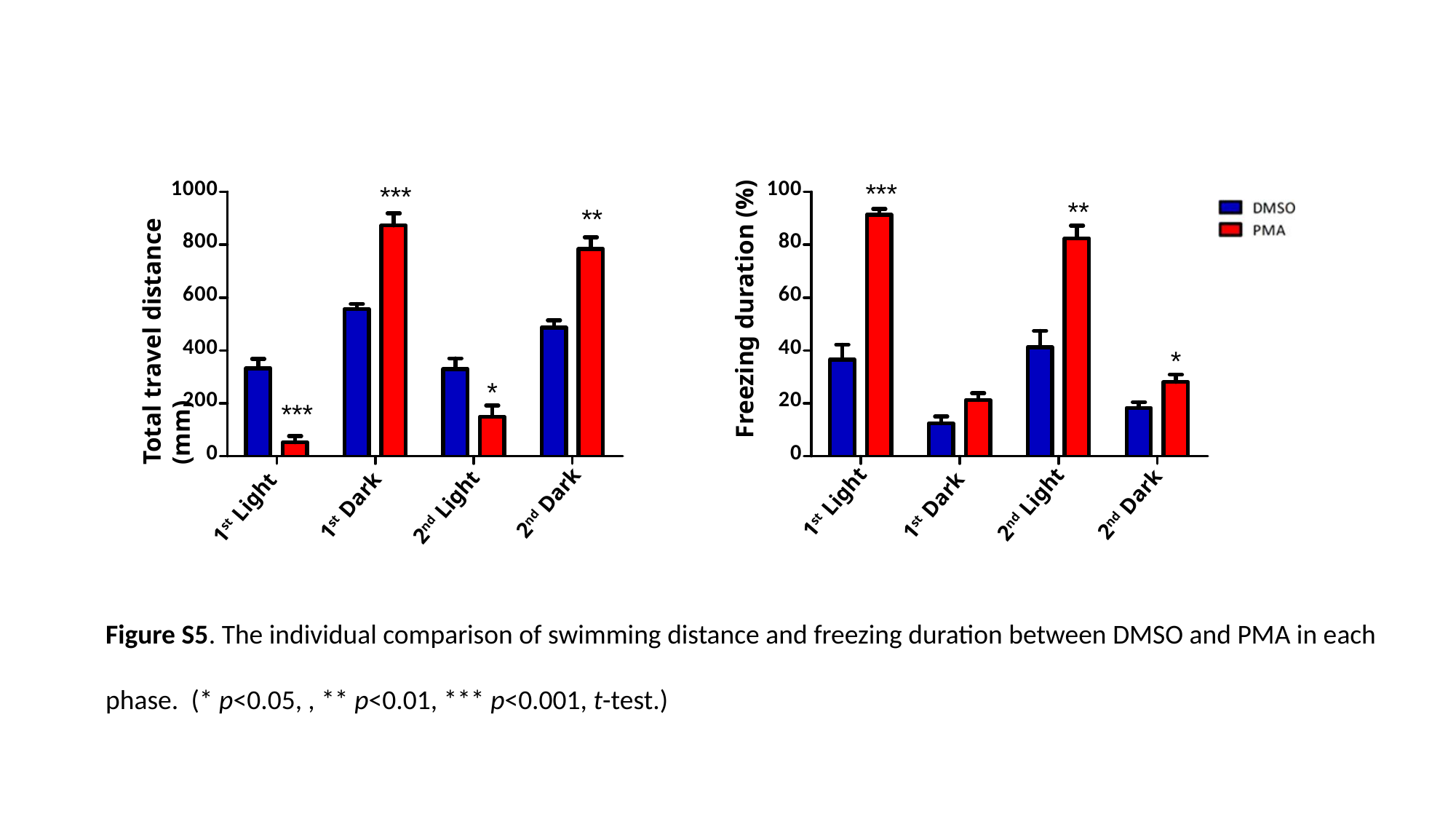

***
***
**
**
Freezing duration (%)
Total travel distance (mm)
*
*
***
1st Light
2nd Dark
2nd Dark
1st Dark
1st Dark
2nd Light
1st Light
2nd Light
Figure S5. The individual comparison of swimming distance and freezing duration between DMSO and PMA in each phase. (* p<0.05, , ** p<0.01, *** p<0.001, t-test.)
