# Supplementary material for: Developmental protein kinase C hyper-activation results in microcephaly and behavioral abnormalities in zebrafish
Source: Transl Psychiatry. 2018 Oct 23;8:232. doi: 10.1038/s41398-018-0285-5 (PMC6199330; doi:10.1038/s41398-018-0285-5)
Supplement: Supplementary file 7 — Supplemental Figure S6 [file 41398_2018_285_MOESM7_ESM.pptx]

## Slide 1
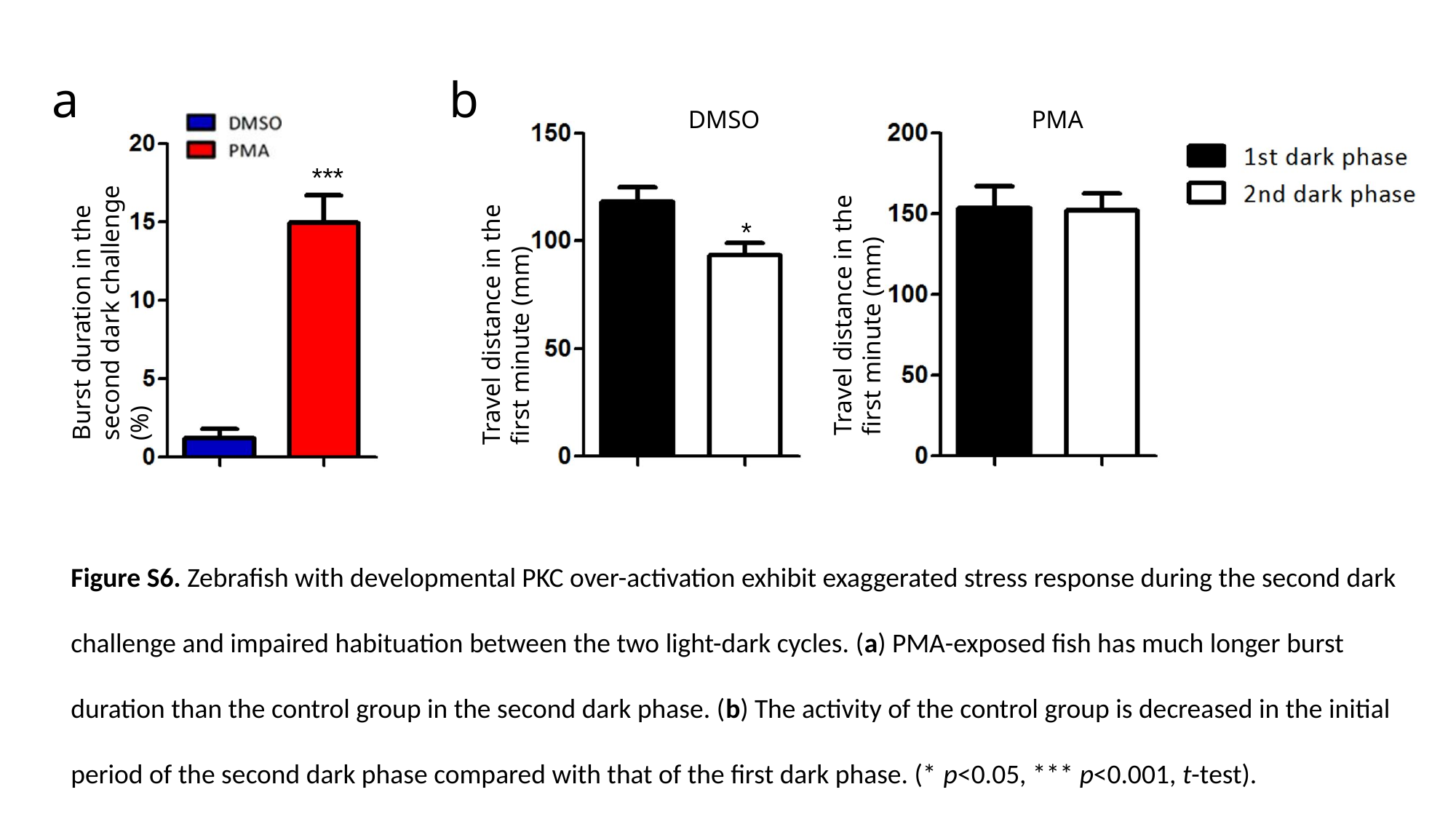

b
a
DMSO
PMA
***
*
Travel distance in the first minute (mm)
Burst duration in the second dark challenge (%)
Travel distance in the first minute (mm)
Figure S6. Zebrafish with developmental PKC over-activation exhibit exaggerated stress response during the second dark challenge and impaired habituation between the two light-dark cycles. (a) PMA-exposed fish has much longer burst duration than the control group in the second dark phase. (b) The activity of the control group is decreased in the initial period of the second dark phase compared with that of the first dark phase. (* p<0.05, *** p<0.001, t-test).
